# Frequency and Molecular Identification of *Cryptosporidium* in Adult Prim’Holstein Dairy Cattle Farms in the North of France

**DOI:** 10.3390/microorganisms12020335

**Published:** 2024-02-05

**Authors:** Gabriela Certad, Nausicaa Gantois, Sophie Merlin, Sophie Martel, Gaël Even, Eric Viscogliosi, Christophe Audebert, Magali Chabé

**Affiliations:** 1 Centre National de la Rrecherche Scientifique (CNRS), Institut National de la Santé et de la Recherche Médicale (Inserm), Centre Hospitalier Universitaire de Lille, Institut Pasteur de Lille, U1019-UMR 9017-CIIL-Centre d’Infection et d’Immunité de Lille, University of Lille, F-59000 Lille, France; nausicaa.gantois@pasteur-lille.fr (N.G.); eric.viscogliosi@pasteur-lille.fr (E.V.); magali.chabe@univ-lille.fr (M.C.); 2 Délégation à la Recherche Clinique et à l’Innovation, Groupement des Hôpitaux de l’Institut Catholique de Lille, F-59000 Lille, France; 3 GD Biotech-Gènes Diffusion, F-59000 Lille, France; s.merlin@genesdiffusion.com (S.M.); s.martel@genesdiffusion.com (S.M.); g.even@genesdiffusion.com (G.E.); c.audebert@genesdiffusion.com (C.A.); 4 PEGASE-Biosciences (Plateforme d’Expertises Génomiques Appliquées aux Sciences Expérimentales), Institut Pasteur de Lille, F-59000 Lille, France

**Keywords:** molecular epidemiology, *Cryptosporidium* infection, adult dairy cattle, France

## Abstract

*Cryptosporidium* apicomplexan protozoa are ubiquitous intracellular agents affecting humans and animals. In particular, bovine cryptosporidiosis is recognized as endemic worldwide. However, epidemiological investigations remain limited in France regarding the burden of these parasites in cattle. To improve our understanding of the epidemiology of cryptosporidiosis, the main aim of this study was to determine the frequency and the genetic diversity of *Cryptosporidium* in adult Prim’Holstein dairy cattle farms in the north of France. Fecal specimens were collected from 1454 non-diarrheic and non-pregnant animals (nulli-, primi-, or multiparous) throughout 20 farms in an area of 110 km around Lille. For *Cryptosporidium* species identification, nested PCR followed by sequence and phylogenetic analyses were used. The overall frequency of *Cryptosporidium* spp. in-fection was 30.00% (C.I. 95%: 12.83–54.33) in farms and 0.89% (C.I. 95%: 0.498–1.57) at the individual level. In primi- or multiparous cows, only *C. andersoni* was found. *C. ryanae*, *C. bovis/xiaoi* and *C. andersoni* were detected in heifers. The phylogenetic tree confirmed that analyzed sequences were grouped with known reference sequences reported in dairy cattle. Further studies on the cumulative prevalence, risks factors and pathogenicity are needed to give a more accurate assessment of the impact of *Cryptosporidium* infection in dairy cattle in France.

## 1. Introduction

*Cryptosporidium* are worldwide intestinal opportunistic protozoan parasites that infect humans as well as a broad spectrum of domestic and wild hosts including ruminants, carnivores, and primates to whom they can cause severe diarrhea [1]. Cryptosporidiosis has been reported in many important livestock species, including cattle, buffaloes, pigs, goats, sheep, horses, camels, donkeys, chickens, and ducks [2]. *Cryptosporidium* parasites have low infective doses as shown in human volunteers [3] and animal models [4], and oocysts are very resistant to environmental and water treatment [1]. Until now, 44 *Cryptosporidium* species and more than 120 genotypes have been recognized [5]. The predominant *Cryptosporidium* species infecting humans are *C. parvum* and *C. hominis*, while *C. bovis*, *C. ryanae*, and *C. andersoni*, together with *C. parvum*, are the causal agents of bovine *Cryptosporidium* infection, even if additional species have also been reported in sporadic cases, such as *C. felis*, *C. hominis*, *C. suis*, *C. canis*, *C. scrofarum*, *C. tyzzeri or C. serpentis* [6]. *C. parvum* has the ability to infect multiple animal hosts, and it is the primary zoonotic cause of cryptosporidiosis [7]. Strikingly, almost 100% of dairy cattle worldwide are infected with *C. parvum* at some point during their lives [5].

The transmission of *Cryptosporidium* from cattle to humans can occur through several routes: the contact with infected animals or carcasses, the consumption of contaminated food derived from beef or dairy cattle and the consumption of crops irrigated with water contaminated with cattle manure [8]. Transmission in animals is mainly caused by the ingestion of oocysts excreted by infected animals, especially newborns in overcrowded facilities [6]. Livestock manure is an important source of infection to both animals and humans, and it has been estimated that the global *Cryptosporidium* load in cattle manure is approximately 3.2 × 10^23^ oocysts per year [6].

The prevalence of *Cryptosporidium* spp. in cattle is age-related. Hence, *C. parvum* is responsible for most infections in pre-weaned calves, while *C. bovis* and *C. ryanae* are the most frequently detected species in post-weaned calves, and *C. andersoni* seems to predominate in heifers and mature cows [9,10,11,12], even if some reports are controversial about this aspect [13]. *Cryptosporidium* infections in neonatal calves are responsible for economic losses mostly associated with the cost of managing the morbidity and mortality of animals. Dehydration, weight loss, retarded growth, and decreased feed efficiency represent some of the severe consequences of cryptosporidiosis in livestock [14]. Currently, no treatment is available against the infection, since most of the tested drugs exhibit only partial prophylactic and therapeutic efficacy in reducing oocyst excretion and disease severity in affected animals [6]. Furthermore, oocysts of *Cryptosporidium* spp. are highly stable in the environment and resistant to almost all conventional disinfection methods and water treatments, making cryptosporidiosis difficult to control [6].

Bovine cryptosporidiosis is recognized as endemic worldwide, even though the prevalence of the parasite varies considerably between geographical areas, animal age, and surveys [6]. In France, the knowledge about the molecular epidemiology of *Cryptosporidium* in cattle remains still limited, and only few publications are available, which are mainly focused on calves. Therefore, the aim of the present study was to provide an update in the molecular epidemiology of *Cryptosporidium* concerning Prim’Holstein, nulli, primi- and multiparous dairy cattle farms in the north of France, being Prim’Holstein the leading French dairy cow, accounting for 66% of livestock. In addition, Hauts-de-France is one of the major French dairy regions, producing 10% of the milk of the country. Overall, 5500 milk producers (i.e., one in five farmers) rank the Hauts-de-France as the fifth French milk producer. Dairy cows in the region includes 300,000 animals, representing 8.4% of the national herd. The average size of a cattle herd is 124 cattle. The herd is relatively concentrated. Nearly 46% of farms own just 16% of the cows, while 54% of farms own 84% of cows [15].

## 2. Materials and Methods

### 2.1. Farm Recruitment

Cattle breeders of the north of France region were contacted directly by researchers and invited to a seminar in order to explain to them the research project and the study procedure as well as request participation in the study. Farmers were eligible for inclusion in the study when the following requirements were satisfied: breeding of cows 100% Prim’Holstein and a minimum of 50 cows in milk production (French average: 56 cows/herd). The farm managers meeting the study criteria who agreed to participate on a voluntary basis and give their written consent were selected for the sampling. All the farms included in the study were commercial ones using the mixed crop–livestock farming system, which combines the cultivation of crops with the rearing of livestock on the same farm and is intended for high-quality safe raw milk production for human consumption. These farms followed strict veterinary periodic controls according to EU hygiene legislation (Regulation 853/2004). Veterinary check outs include controls of the good general state of health of animals, absence of infectious diseases communicable to humans through milk, absence of udder wound likely to affect the milk, control of somatic cells, etc.

### 2.2. Cows Selection

Animal were selected according to the following criteria: adult, Prim’Holstein dairy cattle, nulli-, primi-, or multiparous, asymptomatic and in good general state of health, absence of infectious diseases, without antibiotics treatment for at least 2 months before sampling, non-pregnant or calving at least one month ago. All animals under the study were managed on a semi-extensive system (on pasture for variable periods of their feeding and grazing seasons but indoors during the cold/wet season).

### 2.3. Sampling

Overall, 20 farms in an area of 110 km around the city of Lille in the north of France accepted to participate in the study. The average herd size in these farms during the time frame of the study was 194 (SD, 86) cows (range: 70–409). Animal reproduction technicians from the Gènes Diffusion company (Douai, France) collected a total of 1916 stool samples from 1454 cows from September 2017 to December 2018. To facilitate the collection of a 50 g fecal sample, cows were rectally finger stimulated with sterile-gloved hands. Fecal samples were immediately frozen at −20 °C (or stored at 4 °C for 1 to 2 h before freezing).

### 2.4. Molecular Detection of Cryptosporidium

The NucleoSpin 96 Soil Kit or NucleoSpin Soil Mini Kit (Macherey-Nagel GmbH & Co. KG, Düren, Germany) were used for the DNA extraction from approximately 200 mg of fecal samples according to the manufacturer’s recommendations. DNA was stored at −20 °C until use. The nested PCR targeting the 18S rRNA gene was performed as previously described [16] with slight modifications (the analytical sensitivity of this technique in our laboratory for the detection of *Cryptosporidium* DNA from 5 µL of serial 10-fold 18S rRNA plasmids diluted in a final volume of 50 µL is of 10 copies, which is equivalent to at least 1 oocyst). The external primers used were 5′-TTCTAGAGCTAATACATGCG-3′ (forward) and 5′-CCCATTTCCTTCGAAACAGGA-3′ (reverse). The internal primers used were 5′-GGAAGGGTTGTATTTATTAGATAAAG-3′ (forward) and 5′-AAGGAGTAAGGAACAACCTCCA-3′ (reverse). The first PCR mixture was prepared in a final volume of 50 μL as follows: 10 μL of DNA, 1x HotStarTaq Plus buffer, 2 mM MgCl2, 0.4 μM for each primer, 0.4 μM dNTP each and 1.5 U HotStarTaq Plus DNA polymerase (Qiagen Inc.,Hilden, Germany). The conditions for the PCR were as follows: 94 °C for 5 min, followed by 40 cycles of 94 °C for 45 s, 65 °C for 45 s, and 72 °C for 1 min. The post-extension was completed at 72 °C for 5 min. The second PCR mixture was prepared in a final volume of 50 μL as follows: 2 μL of the primary PCR product, 1x HotStarTaq Plus buffer, 1.5 mM MgCl_2_, 0.4 μM for each primer, 200 μM dNTP each and 1.5 U HotStarTaq Plus DNA polymerase. The nested PCR conditions were the same as those in the first round. Nested PCR reactions were performed in a PTC 200 thermocycler (MJ Research, Waltham, MA, USA).

### 2.5. DNA Sequencing and Analysis

After purification of the positive PCR products, the amplicons were sequenced on both strands (Sanger technology) using the forward and reverse primers of the nested PCR by the company Genoscreen (Pasteur Institute of Lille, Lille, France). MUSCLE in SeaView v4.6 was used for alignment of the sequences [17]. Comparisons with similar sequences of *Cryptosporidium* available on the NCBI server (http://www.ncbi.nlm.nih.gov/BLAST/, accessed on August 2023) using the basic local alignment search tool (BLAST) program were performed. To consider the sequences analyzed in this study as the same *Cryptosporidium* species when compared to references, the identity value should be in the range of 98–100% sequence similarity. All of the nucleotide sequences identified in this study were deposited in GenBank under the accession numbers OR610758 to OR610770.

### 2.6. Phylogenetic Analysis

The SSU rRNA gene sequences (785 bp) obtained in the present study from *Cryptosporidium* spp. isolates were added and aligned to a dataset including reference sequences from *C. andersoni*, *C. baileyi*, *C. bovis*, *C. meleagridis*, *C. occultus*, *C. parvum*, *C. ryanae*, *C. scrofarum*, *C. suis and C. xiaoi* downloaded from the GenBank database. Phylogenetic tree reconstruction was performed using the MEGA X software v. 10.2.6 [18], where the best substitution model was selected using the Bayesian information criterion. Phylogenetic trees were constructed with 1000 replicates, calculating bootstrap values through the maximum likelihood (ML) method and the Tamura 3-parameter model [19]. Neighbor-joining (NJ) and UPGMA trees were also constructed using the MEGA X software program.

### 2.7. Statistical Analysis

The prop.test function in R (version 4.1.1) was utilized to perform a proportion test, allowing the determination of 95% confidence intervals (C.I. 95%) around observed proportions, thereby providing a comprehensive statistical evaluation of the frequency of *Cryptosporidium.*

### 2.8. Ethical Issues

No approval from the Institutional Animal Care and Use Committee or ethics committee was required for this study. Animals in recruited farms were raised following French guidelines for animal care and use. Samples were collected by trained technicians from Gènes Diffusion, holders of the CAFTI (Certificat d’Aptitude aux Fonctions de Technicien d’Insémination-Certificate of Fitness for Insemination Technician Functions), and authorized for biological sampling according to animal welfare. The farmers participating to the study signed an agreement consenting access to animals’ samples for research purposes.

## 3. Results

A total of 1454 animals with a median age of 1025 days (range: 200–4444 days) from 20 farms located in northern France were screened in this survey. Molecular analysis of DNA extracted from animals stools, followed by nested PCR and sequencing, allowed the identification of *Cryptosporidium* spp. in 6 out of 20 (30.00%, C.I. 95%: 12.83–54.33) farms with a frequency ranging between 0 and 3.2% across these farms. The highest number of positive cattle was observed in farm “F12” with 5 samples corresponding to four cows testing positive for *Cryptosporidium* (one cow positive twice). Individually, 13 (0.89%, C.I. 95%: 0.498–1.57) out of 1454 animals were detected as positive (Table 1).

Positive animals ranged in age from 11 to 88 months. Among them, eight were heifers (11 to 33 months of age), four were primi- or multiparous cows (28 to 88 months of age) and one was a heifer at the moment of the first sampling and a cow at the moment of the second sampling (Table 2). In addition, 9 positive samples out of 14 (64%) were collected in the period fall/winter, while the 5 other positive samples (36%) were collected in the period spring/summer. Meanwhile, 5 positive samples out of 14 were collected in the periparturient period of cows (between 1 and 9 weeks after delivery) (Table 2).

**Figure 1:**
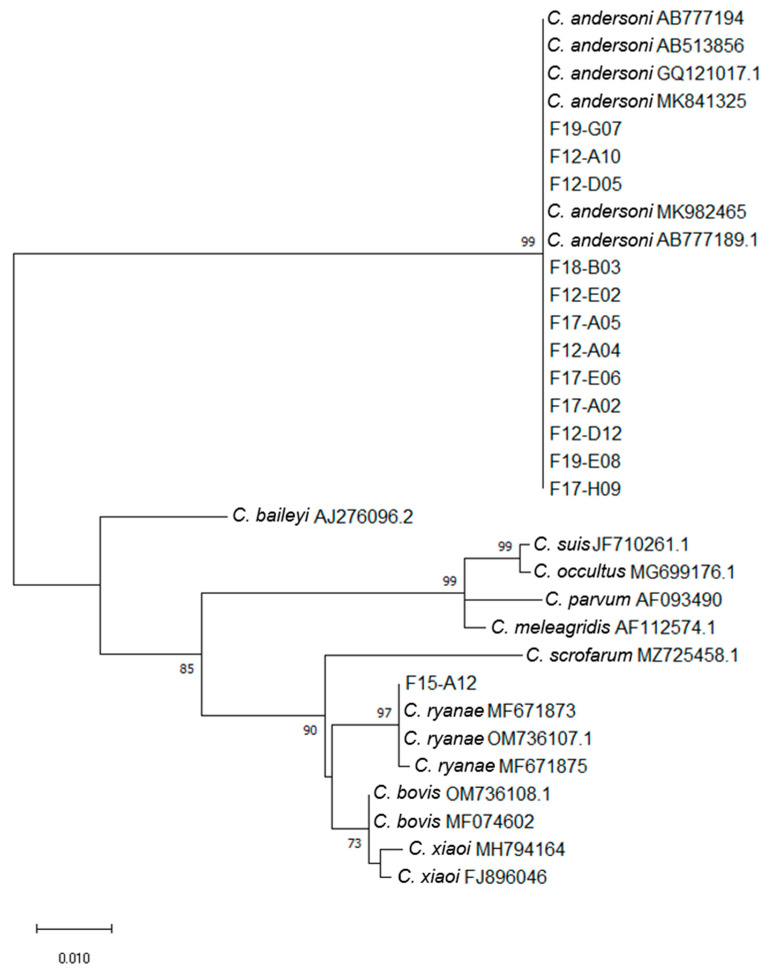
Maximum likelihood phylogenetic analysis of *Cryptosporidium* isolates based on partial SSU rRNA gene sequences (785 bp). Accession numbers of reference sequences of *C. andersoni*, *C. baileyi*, *C. bovis*, *C. meleagridis*, *C. occultus*, *C. parvum*, *C. ryanae*, *C. scrofarum*, *C. suis and C. xiaoi* are indicated. The tree is drawn to scale with branch lengths measured in the number of substitutions per site. Numbers near the individual nodes indicate bootstrap values (%) above 50% (1000 replicates). Neighbor joining and UPGMA methods lead to similar topologies; only the maximum likelihood tree is shown.

Sequence and phylogenetic analyses at the 18S rRNA gene locus identified 3 *Cryptosporidium* species among the 14 nested PCR positive samples: *C. andersoni* (*n* = 12), *C. ryanae* (*n* = 1) and *C. bovis/xiaoi* (*n* = 1) (Figure 1 and Table 1 and Table 2). Most *C. andersoni* sequences were 100% homologous to MK982465, while three *C. andersoni* sequences (i.e., F19-G07, F12-A10 and F12-D05) were identical to MK841325. These three sequences could only be distinguished from the other nine by the deletion of a “T” at position 417 of the sequence. All the *C. andersoni* sequences obtained in this study were deposited in GenBank under the accession numbers OR610758–OR610759 and OR610761–OR610770. One sample (F15-A12) was identified as *C. ryanae* and showed 100% identity with the MF671873 sequence (found in bovine stools in China). This sequence differed by 1 bp (T-for-G substitution at position 677) from MF671875 (sequence deposited by the same authors at the same time and also found in bovine stools in China). The *C. ryanae* sequence found in our study has been deposited in GenBank under accession number OR610760. The complete sequence of the nested PCR product could not be obtained for the last sample F20-B09. However, the reverse sequence of this sample showed 100% identity with the sequence of *C. bovis* MFO74602, while the forward sequence showed 100% identity with the sequences of *C. bovis* MFO74602 and *C. xiaoi* FJ896046.

No farm was found positive for both *C. andersoni* and *C. ryanae* (Table 1 and Table 2). However, both *C. andersoni* sequences having as little 1 bp difference over the nested-PCR amplicon (identical to Genbank accession numbers MK841325 or MK982465) were found on the same farm. Thus, on farm F12, the sequence identical to Genbank accession number MK841325 was found in two cows, and the sequence identical to Genbank accession number MK982465 was found in the other three cows. The two positive cows from farm F19 each carried an isolate identical to MK841325 or MK982465. Finally, the MK982465 sequence was the only one found in the four positive cows from farm F17.

To note, the same *C. andersoni* sequence (MK982465) was found in both samples F12-D12 and F12-A04, which came from the same animal at two different times.

In Prim’Holstein cows, only *C. andersoni* was found. *C. ryanae*, *C. bovis/xiaoi* and *C. andersoni* were detected in heifers. The *C. bovis/xiaoi* and *C. ryanae* positive heifers were 11 and 19 months old, respectively.

## 4. Discussion

Overall, *Cryptosporidium* frequency on screened farms in the current study was 30%. This frequency is similar to that found in a Canadian national survey among post-weaned calves and adult dairy cattle [20]. Interestingly, *Cryptosporidium* prevalence in cattle seems to be higher in industrialized countries when compared to non-industrialized ones due to the animal production intensity, which is more developed in the first ones [20,21]. At the individual level, the observed frequency of *Cryptosporidium* spp. in cattle was 0.89% (13/1454). Even if this frequency seems low, previous reports on the prevalence of the parasite in adult cattle have indicated that infection rates range from 0 to 71%, although most reported prevalence reached less than 7% [22].

The low infection rate is probably explained among other factors by the animal age. Indeed, several studies about bovine *Cryptosporidium* infection have reported a host age related susceptibility being the infection more frequent in pre-weaned calves (<8 weeks) [11,23,24]. Animals included in this survey were adults with an average age of 1025 days. Comparable reports in Europe have described a prevalence of less than 10% in healthy adult cows in Spain, Belgium, the Netherlands or France with infection rates of 10%, 6%, 2% and 4%, respectively [13,25]. The decrease in prevalence of *Cryptosporidium* infection in dairy cattle related to age is likely due to the development of immunity from previous exposure/infection [19]. In addition, a low intensity of oocyst excretion without clinical manifestations has been described in both adult beef and dairy cattle [26].

Concerning the species identified in the current study, all of them have been reported in cattle worldwide [5] and in particular in France (Table 3). According to the phylogenetic tree, the sequences obtained in this study were grouped with known reference sequences already reported in dairy cattle. No new sequences were found. In addition, an age-related pattern of species distribution in bovine cryptosporidiosis has also been described in which *C. parvum* is the species most frequently found in calves, causing most of the *Cryptosporidium* infections in less than 5-week-old calves. Then, when animals are older, they can be infected successively with *C. ryanae*, *C. bovis* and *C. andersoni*, as it has been reported in France (Table 3) and also in other countries such as Brazil, China and India [23,24,27,28]. Consistently, in the current survey, a *Cryptosporidium* spp. age-related trend with an increase in the occurrence of *C. andersoni* and *C. ryanae* and an absence of *C. parvum* infection according to the increasing age of animals was observed (Table 3).

However, this disagrees with recent findings reporting *C. parvum* among the dominant species infecting cows in the Netherlands and Belgium [13]. In France, variations in the distribution of *Cryptosporidium* species in different studies are shown in Table 3. In a previous survey in the Hauts-de-France, it was reported that the most predominant species (42.8%) in adult dairy cattle was *C. bovis* [13], while in a follow-up performed in the same area by the same group, *C. andersoni* was the most prevalent species (57.14%) followed by *C. bovis* (28.57%) [31]. Different factors may explain this difference in prevalence and species distribution in the same country such as sampling size, type of breed, farm management practices (intensive, semi-intensive or extensive), farm location (urban/rural) or feeding system among others [5,32].

In the current work, most of the *Cryptosporidium* infection cases were detected in the winter/fall period, suggesting a trend between the occurrence of *Cryptosporidium* infection and seasons. Variations between seasons might be attributed to particular climatic situations more frequent in winter including heavy rains, snow melting and floods, which can cause sewage overflow and increase agricultural runoff, favoring the survival and dissemination of oocysts [33]. However, due to the semi-intensive system applied by the participating farms, animals are placed indoors and in cohousing during the autumn and winter seasons. Probably, this practice could facilitate promiscuity and inter-animal contamination. On the other hand, a low prevalence of *Cryptosporidium* has already been reported in cattle under extensive management systems, which may be due to lower exposure to infection. In fact, in extensive systems, oocysts are dispersed on a large surface and have a reduce viability due to direct sunlight exposure [34]. Further studies have to be conducted to confirm this observation. It was also observed in the current study that 35% of Cryptosporidium-positive cows were in the periparturient period and they were all infected by *C. andersoni*. The periparturient rise in *C. andersoni* has already been documented prior to this study [35].

## 5. Limitations of the Study

One of the main limitations of this kind of study relates to the methods used for detection of the infection. Indeed, a lower prevalence of *Cryptosporidium* in cows could be explained by technical problems concerning detection. Although molecular methods are more sensitive than standard microscopic observation for the detection of *Cryptosporidium*, they may not be enough when processing voluminous fecal samples from adult cows, thereby diluting protozoan oocysts [22]. Accordingly, a method of *Cryptosporidium* detection adapted to adult cattle samples was developed by performing concentration and flotation before molecular screening, showing in this way an increase in *Cryptosporidium* detection [22]. Additionally, only single stool sample tests were performed for the majority of animals; therefore, the prevalence was probably underestimated. Consequently, more sensitive methods appropriate to adult cattle fecal processing as well as the use of internal controls to identify the presence of PCR inhibitors would be required. Moreover, the study attempted to show the association of *Cryptosporidium* infection with age and seasonality. However, it was based on limited number of positive cases. 

## 6. Conclusions

In conclusion, this study had the largest sampling ever carried out for the study of *Cryptosporidium* of cattle, providing data on the infection rate of the parasite in adult dairy cattle in France. Interestingly, results showed that even if participating farms have strict measures of hygiene control and strict veterinary periodic visits, the parasite circulates confirming that this infection is difficult to control due to environmentally stable oocysts resistant to many disinfectants, low infective dose, absence of vaccines and limited treatment options [36].

The absence of *C. parvum* infection, the major zoonotic species, suggests that adult dairy cattle represent a low risk source of infection for humans even if some of the *Cryptosporidium* spp. detected in cattle such as *C. andersoni* or *C. bovis* have been reported infecting humans too [20]. Although infections with the latter two Cryptosporidium species are generally asymptomatic in cattle, it has been reported that chronic *C. andersoni* infections in these animals may result in gastritis associated with reduced milk production and poor weight gain, with potential clinical and economic impact [37], and it may also contribute to environmental contamination [20]. Further studies on cumulative prevalence, risks factors and pathogenicity are required to give a more accurate assessment of the impact of Cryptosporidium infection in dairy cattle in France.

## Figures and Tables

**Table 1:** Cryptosporidium infection rate and species identification according to farms.

Farms Identification	N° of Animals/Farm	N° of Tested Samples/Farm	N° of *Cryptosporidium* Positive Animals (%)	95% Confidence Intervals (C.I. 95%)	*Cryptosporidium* Species
F1	249	205	0	NA ^a^	NA
F2	154	136	0	NA	NA
F3	204	137	0	NA	NA
F4	245	96	0	NA	NA
F5	153	117	0	NA	NA
F6	82	54	0	NA	NA
F7	70	33	0	NA	NA
F8	136	11	0	NA	NA
F9	244	60	0	NA	NA
F10	112	24	0	NA	NA
F11	219	71	0	NA	NA
F12	208	157	5 (3.18)	1.17–7.66	*C. andersoni*
F13	409	128	0	NA	NA
F14	312	43	0	NA	NA
F15	110	55	1 (1.81)	0.10–11.00	*C. ryanae*
F16	233	67	0	NA	NA
F17	230	149	4 (2.68)	0.86–7.16	*C. andersoni*
F18	123	112	1 (0.89)	0.47–5.60	*C. andersoni*
F19	275	166	2 (1.20)	0.21–4-74	*C. andersoni*
F20	107	95	1 (1.05)	0.6–6.56	*C. bovis/xiaoi*
Total	3875	1916	14 (0.73)	0.42–1.26	*C. andersoni*, *C. ryanae*, *C. bovis/xiaoi*

^a^ NA, not applicable.

**Table 2:** Description of positive samples according to animal age, delivery, sampling season and Cryptosporidium species detection identified at the SSU rRNA gene locus.

Sample Identification	Age in Months (Age Class) ^c^	Number of Delivery	Weeks for Sample Collection after Last Delivery	Sampling Season	*Cryptosporidium* Species	Gene Accession Number	% of Identity with Reference Sequence
F17-E06	21 (heifer)	0	NA ^a^	Fall	*C. andersoni*	OR610758	100 (MK982465.1)
F20-B09	11 (heifer)	0	NA	Fall	*C. bovis/xiaoi*	-	100/forward (MF074602/FJ896046) 100/reverse (MF074602)
F19-E08	29 (cow)	1	4	Winter	*C. andersoni*	OR610759	100 (MK982465.1)
F15-A12	19 (heifer)	0	NA	Fall	*C. ryanae*	OR610760	100 (MF671873)
F17-H09	18 (heifer)	0	NA	Winter	*C. andersoni*	OR610761	100 (MK982465.1)
F17-A02	88 (cow)	5	8	Fall	*C. andersoni*	OR610762	100 (MK982465.1)
F19-G07	28 (cow)	1	1	Winter	*C. andersoni*	OR610763	100 (MK841325.1)
F12-A04 ^b^	29 (heifer)	0	NA	Spring	*C. andersoni*	OR610764	100 (MK982465.1)
F17-A05	46 (cow)	2	9	Summer	*C. andersoni*	OR610765	100 (MK982465.1)
F18-B03	24 (heifer)	0	NA	Summer	*C. andersoni*	OR610766	100 (MK982465.1)
F12-A10	33 (heifer)	0	NA	Spring	*C. andersoni*	OR610767	100 (MK841325.1)
F12-D12 ^b^	31 (cow)	1	5	Spring	*C. andersoni*	OR610768	100 (MK982465.1)
F12-E02	25 (heifer)	0	NA	Fall	*C. andersoni*	OR610769	100 (MK982465.1)
F12-D05	20 (heifer)	0	NA	Fall	*C. andersoni*	OR610770	100 (MK841325.1)

^a^ NA, not applicable; ^b^ These two samples are from the same animal tested at different moments; ^c^ Age class: heifer: female adult cattle who has not produced a calf; cow: female adult cattle who has produced at least one calf.

**Table 3:** Age related Cryptosporidium distribution in cattle from France according to different studies.

Localization	Method of Detection	Age of Animals	*C. parvum*		*C. ryanae*		*C. bovis*		*C. xiaoi*		*C. andersoni*		ND		References
			N° of Positive	Prevalence (%)	N° of Positive	Prevalence	N° of Positive	Prevalence	N° of Positive	Prevalence	N° of Positive	Prevalence	N° of Positive	Prevalence	
Britanny	PCR and sequencing	5 weeks	59/68	87	3/68	4	1/68	2	0	0	0	0	5/68	7%	[23]
		15 weeks	1/59	1.69	26/59	44	27/59	45	0	0	0	0	5/59	9%	
		22 weeks	0/20	0	10/20	50	9/20	45	0	0	0	0	1/20	5%	
Normandy	PCR and sequencing	<21 days	80/82	97.6	0	0	2/82	2.4	0	0	0	0	2/82	0	[29]
Allier Ardèche, Côte-d’Or, Moselle, Saône et-Loire, Yonne	PCR and sequencing	≤45 days	29/31	93.54	0	0	0	0	0	0	0	0	0	0	[30]
Hauts-de-France	PCR and sequencing	<3 months	49/72	68.1	3/72	4.2	19/72	26.4	1/72	14.3	0	0	0	0	[13]
		Adult ^a^	2/7	28.6	1/7	14.3	3/7	42.8	0	0	1/7	14.3	0	0	
Hauts-de-France	PCR and sequencing	<3 months	28/38	73.6	4/38	10.52	5/38	13.15	0	0	1/38	2.63	0	0	[29]
		Adult ^a^	1/7	14.28	0	0	2/7	28.57	0	0	4/7	57.14	0	0	
Hauts-de-France	PCR and sequencing	11–33 months	0	0	1/9	11.11	0	0	0	0	7/9	77.78	1/9	11.11	Present study
		28–88 months	0	0	0	0	0	0	0	0	5/5	100	0	0	

^a^ Age of adult cows was not indicated.

## Data Availability

The sequences obtained in the study were deposited in GenBank under the accession numbers (OR610758–OR610770).
